# Supplementary material for: Low-loss YIG-based magnonic crystals with large tunable bandgaps
Source: Nat Commun. 2018 Dec 21;9:5445. doi: 10.1038/s41467-018-07893-5 (PMC6303406; doi:10.1038/s41467-018-07893-5)
Supplement: Supplementary file 1 — Supplementary Information [file 41467_2018_7893_MOESM1_ESM.pdf]

Low-loss YIG-based magnonic crystals with large tunable bandgaps

Huajun Qin[1*], Gert-Jan Both[1,2], Sampo J. Hämäläinen[1], Lide Yao[1] & Sebastiaan van Dijken[1*]

[1]*NanoSpin, Department of Applied Physics, Aalto University School of Science, FI-00076Aalto, Finland*

[2]*Department of Applied Physics, Eindhoven University of Technology, 5600 MB Eindhoven, Netherlands*

[*] huajun.qin@aalto.fi, sebastiaan.van.dijken@aalto.fi

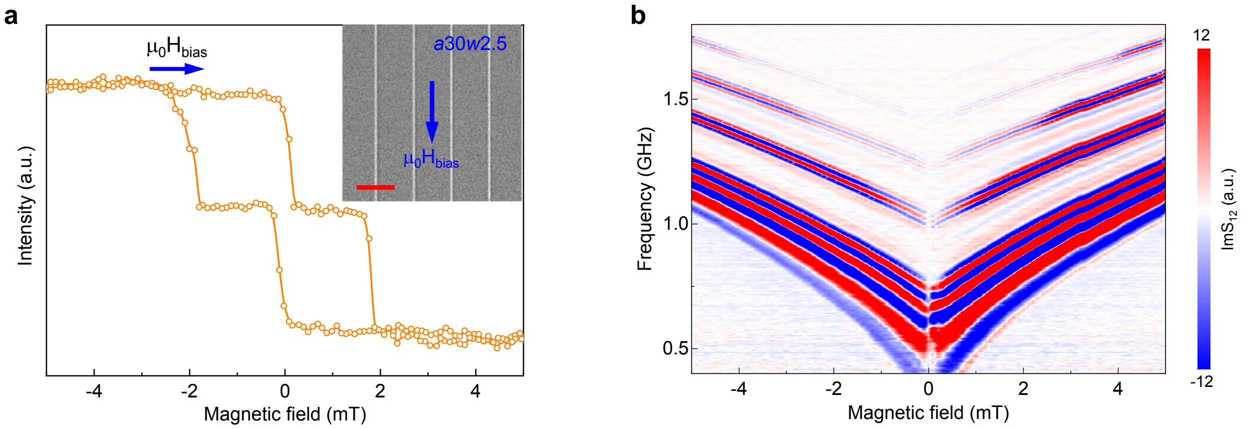

Supplementary Figure 1. **a**, Magneto-optical Kerr effect hysteresis curve showing independent magnetic switching in the YIG and CoFeB stripes of a $a30w2.5$ magnonic crystal. The scale bar in the Kerr microscopy image corresponds to 30 μm. **b**, Imaginary part of S_{12} as a function of external magnetic bias fields. The absence of discontinuities in the imaginary part of S_{12} demonstrates that switching between antiparallel and parallel magnetization configurations at -2.0 mT or $+2.0$ mT does not effect the transmission of spin waves. We attribute this invariance to the large difference in YIG and CoFeB stripe width and the relatively small precession of magnetization in CoFeB at $1.0 - 1.8$ GHz.

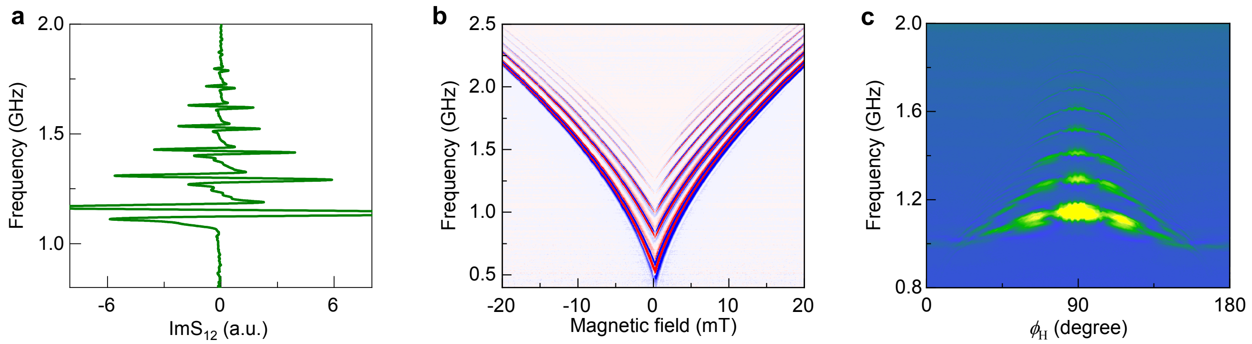

Supplementary Figure 2. **a**, Spin-wave transmission spectrum (imaginary part of S_{12}) for a $a50w5$ CoFeB/YIG magnonic crystal at a magnetic bias field of 5 mT. **b**, Imaginary part of S_{12} for the same crystal at different external magnetic bias fields. **c**, Dependence of spin-wave transmission spectra (amplitude of S_{12}) on magnetic field angle in a $a50w5$ CoFeB/YIG crystal. In this graph, 90° corresponds to the excitation of DE spin waves. The data in this figure complement the results for a $a30w2.5$ CoFeB/YIG magnonic crystal in Figs. 2b-d of the main manuscript.

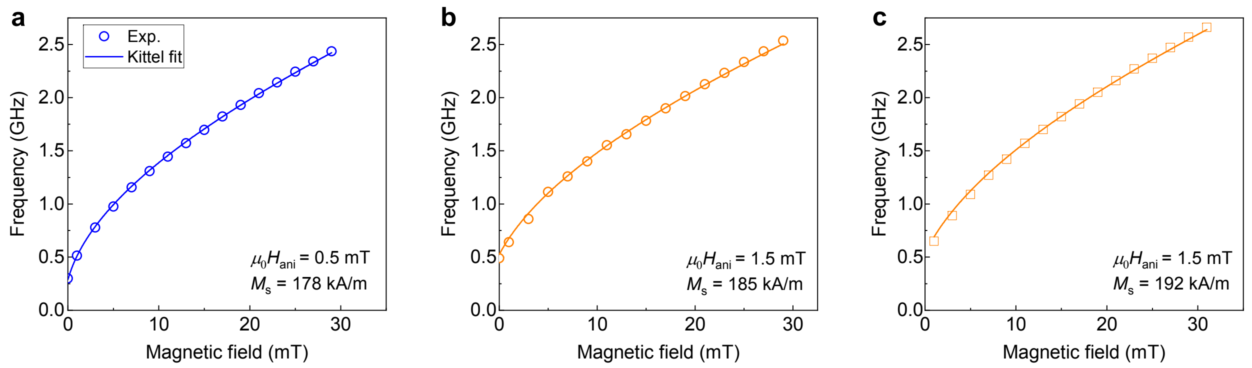

Supplementary Figure 3. **a-c**, Kittel-formula fits to experimental FMR data for (a) a continuous YIG film, (b) a $a30w2.5$ CoFeB/YIG crystal, and (c) a $a30w5$ CoFeB/YIG crystal. The measurements are performed in transmission. Fitting parameters are given in the graphs. Derived values of $\mu_0 H_{\rm ani}$ and $M_{\rm s}$ for CoFeB/YIG crystals (plotted in (b) and (c)) are used as inputs for spin-wave dispersion calculations. The calculation results are shown in Figs. 3d-f of the main manuscript.

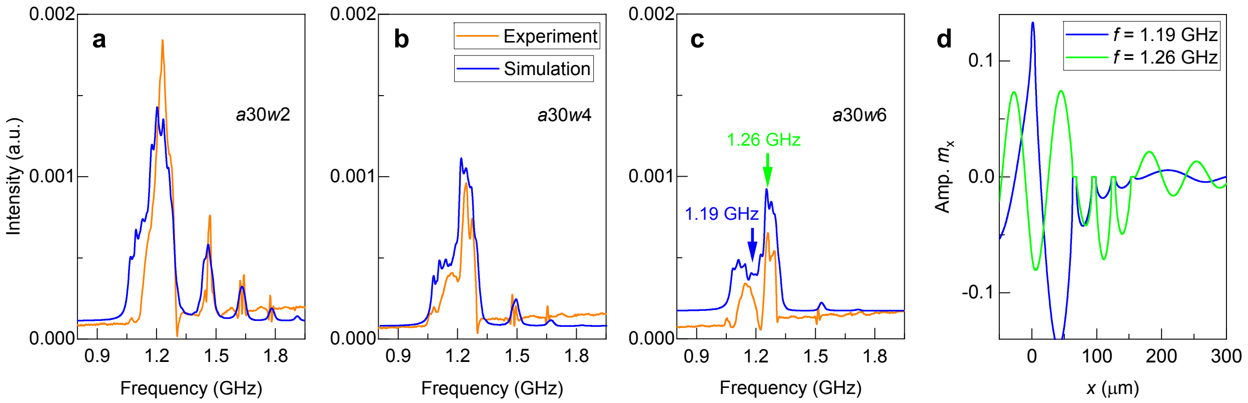

Supplementary Figure 4. **a-c**, Experimental and simulated spin-wave transmission spectra for YIG magnonic crystals with $a = 30$ µm and $N = 4$ airgaps of varying width (w) For $w = 4$ µm and $w = 6$ µm, an extra feature at $\sim$1.2 GHz indicates a suppression of spin-wave transmission in the lowest frequency pass band. **d**, Micromagnetic simulations of the spin-wave amplitude in the $a30w6$ crystal clarifying the origin of spin-wave suppression. The displayed curves are simulated at $f = 1.19$ GHz and $f = 1.26$ GHz. At $f = 1.19$ GHz, i.e., the frequency corresponding to the extra feature in the first allowed miniband, the amplitude of spin waves is reduced. The wavelength of excited spin waves at this frequency equals the total length of the magnonic crystal. At $f = 1.26$ GHz, the wavelength of spin waves does not match the crystal size and the amplitude of transmitted spin waves is larger.

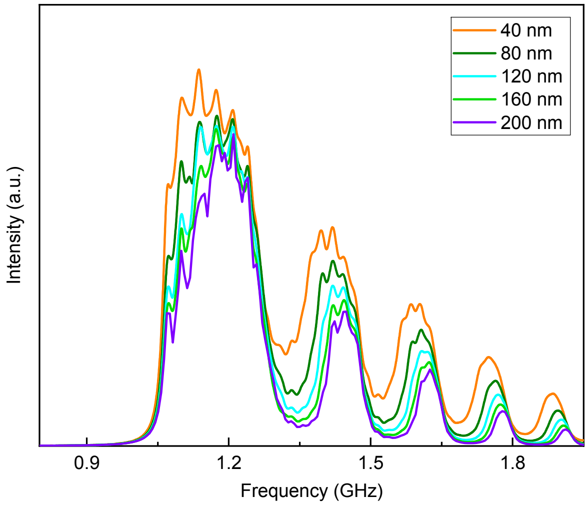

Supplementary Figure 5. Simulated spin-wave transmission spectra for different non-magnetic layer thicknesses at the CoFeB/YIG interfaces. Because a spacer layer of 0.16 μm reproduced the experimental transmission spectra best, we performed all simulations in the main manuscript with this parameter. The data indicate that a reduction of the non-magnetic interface layer increases the transmission of spin waves in the allowed minibands and decreases the size and depth of the bandgaps. In experiments, a non-magnetic interface layer is most likely produced by argon-ion milling of the YIG film.

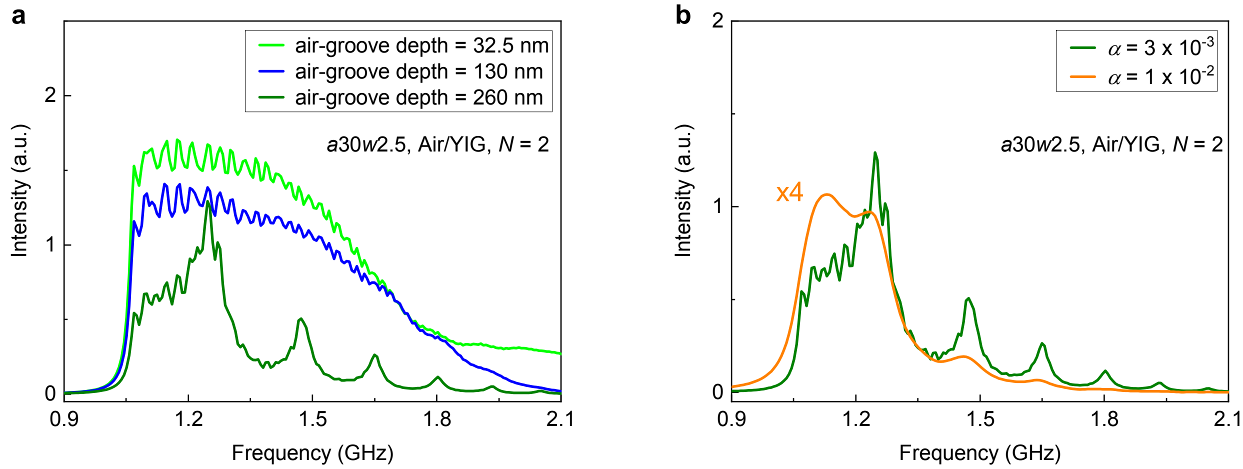

Supplementary Figure 6. **a**, Simulated spin-wave transmission spectra for a 260-nm-thick YIG film with $N = 2$ air grooves. The depth of the grooves is 32.5 nm, 130 nm, and 260 nm, respectively. Only if the grooves extend throughout the entire film, $N = 2$ provides sufficient Bragg scattering to open up bandgaps. Bandgap formation in YIG films with more shallow grooves requires a larger number of scattering units. The damping parameter in these simulations is 0.003. **b**, Simulated spin-wave transmission spectra for $a30w2.5$ crystals with $N = 2$ airgaps. The damping parameter in the two simulations is set to 0.003 and 0.01. The spectrum for $\alpha = 0.01$ is multiplied by a factor 4 to facilitate direct comparisons of the intensity modulations. The simulations demonstrate that the opening of bandgaps with a very limited number of airgaps is not possible in materials with stronger magnetic damping (e.g. ferromagnetic metals). Moreover, ultralow damping in YIG enhances the transmission of spin waves in allowed minibands.
